# An approach to measure compliance to clinical guidelines in psychiatric care

**DOI:** 10.1186/1471-244X-8-64

**Published:** 2008-07-25

**Authors:** Tord Forsner, Anna Åberg Wistedt, Mats Brommels, Yvonne Forsell

**Affiliations:** 1 Department of Public Health Sciences, Karolinska Institutet, Stockholm, SE-171 76, Sweden; 2 Department of Clinical Neuroscience, Section of Psychiatry St Göran's Hospital, Karolinska Institutet, Stockholm, SE-112 81, Sweden; 3 Medical Management Centre, Department of Learning, Informatics, Management and Ethics, Karolinska Institutet, Stockholm, SE-171 77 Sweden; 4 Department of Public Health, University of Helsinki, Helsinki, Finland

## Abstract

**Background:**

The aim of this study was to measure six months compliance to Swedish clinical guidelines in psychiatric care after an active supported implementation process, using structured measures derived from the guidelines.

**Methods:**

In this observational study four psychiatric clinics each participated in active implementation of the clinical guidelines for the assessment and treatment of depression and guidelines for assessment and treatment of patients with suicidal behaviours developed by The Stockholm Medical Advisory Board for Psychiatry. The implementation programme included seminars, local implementation teams, regular feedback and academic visits. Additionally two clinics only received the guidelines and served as controls. Compliance to guidelines was measured using indicators, which operationalised requirements of preferred clinical practice. 725 patient records were included, 365 before the implementation and 360 six months after.

**Results:**

Analyses of indicators registered showed that the actively implementing clinics significantly improved their compliance to the guidelines. The total score differed significantly between implementation clinics and control clinics for management of depression (mean scores 9.5 (1.3) versus 5.0 (1.5), p < 0.001) as well as for the management of suicide (mean scores 8.1 (2.3) versus 4.5 (1.9), p < 0.001). No changes were found in the control clinics and only one of the OR was significant.

**Conclusion:**

Compliance to clinical guidelines measured by process indicators of required clinical practice was enhanced by an active implementation.

## Background

Interest in evidence-based medicine (EBM) has grown exponentially. The focus has been on helping clinicians, patients and policy maker to use the best scientific evidence in their decision-making [1]. Several studies stress the difficulties of implementing EBM and the challenges of achieving performance change in health care [2-4].

Psychiatric care is changing more rapidly than other areas of medicine [5]. Variations in the quality of psychiatric care, for example in depression treatment, have been described in psychiatric practice, including gaps between clinical practice and evidence-based guideline recommendations [6,7]. Clinical guidelines are useful tools to increase the use of EBM and to implement research findings [8-10]. Successfully implementing clinical guidelines and changing practices to reflect current evidence is an important goal and efficient tools are needed to achieve that goal [11]. There is also a need of continuous feedback and formal evaluation of guidelines compliance. One way of evaluating guidelines implementation is to use indicators derived from the clinical guidelines as measures. When those represent best practice as prescribed by the literature, or the consensus view of experts, it can be argued that they also are process measures of the quality of care. As such, guidelines based indicators are a powerful tool of monitoring care and identifying areas of clinical care needing improvement [12]. A recent systematic review by Weinmann et al. [13] emphasised that there is only a small number of implementation studies of psychiatric guidelines. The effects of the guidelines and role of involvement on improvement were reported to be moderate or limited. Complex multifaceted interventions, specific psychological methods, feedback and ongoing support were associated with a more positive outcome [13].

Health care is an important part of the Swedish welfare system and the health service act states that all citizens should have equal access to health care services, regardless of where they live or their financial situation. Regional health authorities, the County Councils, own and operate nearly all hospital and primary care. In the Stockholm County, representatives of public purchasers and providers meet in the Stockholm Medical Advisory Board in order to develop regional clinical guidelines. The shared aim is to provide high quality care on equal terms for all county citizens [14,15]. The Stockholm Medical Advisory Board for Psychiatry in the medical advisory organisation has developed clinical guidelines for different psychiatric disorders with the intention to require those to be implemented in all psychiatric clinics in the Stockholm County. After the publication of the clinical guideline for depressive disorders in 2003, a pilot study was conducted in order to monitor the implementation. An implementation programme was initiated based on the regular assessment of outcome and process quality parameters, defined as above, repeated benchmarking and feedback mechanisms. The implementation process was introduced and found feasible. This study reports the findings of a quasi-experiment involving observations before-and-after the active implementation of clinical guidelines for the care of depression patients and suicidal patients with a comparison group. Guidelines compliance was assessed by identifying predefined measures of requirements of good practice in patient records.

## Methods

The hypothesis to be tested were: the overall depression care quality score is higher for patients treated in implementation clinics than in control clinics and the overall suicidality care quality score is higher for patients treated in implementation clinics than in control clinics.

### Participating clinics

There were six psychiatric departments in Stockholm County at the time of the study. Before the study started all heads of psychiatric departments were invited and participated in an implementation conference in Stockholm. Four departments decided to participate in the study, two in the implementation of guidelines for depression and two for the guidelines for suicidal behaviours. Six psychiatric clinics from the four departments were included. Two of the clinics only received the guidelines and served as controls. The duration of the study was six months.

### Implementation process at the four intervention clinics

The implementation started with a series of seminars based on the clinical guidelines that engaged all staff members. The psychiatrists who had taken part in the design of the guidelines led the seminars. At each of the four clinics local multidisciplinary teams of nurses, physicians, counsellors and psychologists were established. Two of the clinics implemented the clinical guidelines for assessment and treatment of depression and two clinics implemented the guidelines for assessment and treatment of patient with suicidal behaviours. The teams set local goals and identified needs of education and training after analyzing the gap between clinical guidelines and ongoing practice. During the process these goals were evaluated at regular meetings and the clinical guidelines were refined. A number of indicators operationalising the guidelines' requirements on good clinical practice were defined. The presence of those indicators in the patient records were documented and served as measures of guidelines adherence. An internal benchmarking using the registered data was performed at regular intervals. The clinics reported their results and they were compared to averages of the other three intervention clinics. The first author made regular visits to the clinics during the six months period. In summary the implementation programme included multifaceted interventions, seminars, local implementation teams, regular feedback and academic visits.

### Implementation at the two control clinics

The guidelines were also distributed to the comparison clinics, but no seminars were conducted and no local teams were established.

### Included medical records

Patient records from adult men and women, who had an ICD-10 or DSM-IV diagnose of depression were eligible for inclusion in the study on the implementation of the clinical guidelines for depression [16,17]. Patient records were randomly selected. From the two intervention clinics 122 records were selected before and 121 records six months after the start of the implementation process. At the control clinics 61 records were selected before and 60 records after. For the implementation of the clinical guidelines for suicide attempters the inclusion criteria were patient records from adult men and women, which were appraised at a psychiatric emergency clinic after a suicide attempt. From the two intervention clinics 121 records were selected before and 120 records six months after the start of the implementation process. At the control clinics 61 records were selected before and 60 records after. The inter-rater reliability revealed a Cohen's Kappa statistics of 0.92–1.0, lowest for the assessment of the documentation of treatment plan (care plan).

### Measure of compliance

Requirements of recommended practice included in the regional clinical guidelines for depression and for suicide attempters released in 2002 and 2003 were used as indicators of compliance [14,15]. Process indicators developed to assess the quality of care for depression and for suicidal patient were used. To assess whether the requirements were met (and thus the indicator "present") when interpreting notes in the medical records a modified audit instrument by Gardulf and Nordström [18] was used. Absence of data was assumed to indicate that the relevant activities had not been undertaken. Presence of an indicator was given a score from 0–2, (0 = absence, 1 = present but not exactly documented according to the definition and 2 = a clear occurrence). For example a score of one was given if the suicide assessment were not structured according to definition and fulfilled the criteria. The scale was dichotomised into 0 and 1 (1 and 2). Total score for the guidelines for treatment of depression were 11 points and 13 for the guidelines for treatment of suicidal behaviours.

### Indicators from the clinical guidelines for depression

• The time between referral and contact is recorded.

• Diagnostic assessment: the medical record should include at least three of the DSM-IV symptoms for Major Depression [17].

• Standardized rating scale: clinical depression assessment performed using a standardized rating scale.

• Diagnostic structured instrument documented, e.g. Structured Clinical Interview for DSM-IV-TR (SCID) [19].

• The use of a standardized rating scale during treatment for assessment of symptoms and behaviour documented.

• Substance/drug abuse assessment performed using screening instrument e.g. The Alcohol Use Disorders Identification Test (AUDIT) [20].

• Treatment plan (care plan) documented.

• Evaluation of outcome included, e.g. documentation of whether the patient had responded to antidepressive treatment, achieved symptom remission or reduction of symptoms between admission and follow-up.

• Continuity: ability to provide uninterrupted care over time measured as numbers of clinicians involved per patient episode.

• Structured suicide assessment using a standardized rating scale documented.

• Antidepressant medication documented, started at the second visit.

### Indicators from the clinical guidelines for suicide attempters

All indicators listed above, and the following additional:

• Specialist assessment: a documented assessment done by a senior physician within 24 hours after the suicide attempt.

• Follow-up assessments performed.

• Evaluation assessment after discharge documented.

### Data collection

Staff from the participating local teams at each clinic reviewed the medical records and documented the presence of the indicators of compliance. The first author instructed them and a consensus meeting was held, including a calibrating process. The first author used a random replicate sample of 40 medical records to assess inter-rater reliability. In addition, the staff doing reviews received regular tutoring. The study took place over a six-month period and the first data collection was performed in May 2003, before the implementation. The second data collection took place in November 2003. Data from administrative information system were used to identify records that fulfilled the inclusion criteria, and samples from each clinic were selected at random dates during the study period. This study were approved by The Central Ethical Review Board at Karolinska Institutet.

### Statistical analysis

The data were analysed using the SPSS for Windows, Version 15. The inter-rater reliability was analysed by calculating Cohen's Kappa. The statistical significances of the differences before and after the implementation were calculated using Chi-square tests. Associations between age, gender and percentages of patients being treated in accordance with each indicator was analysed using Pearson correlation tests. Age and gender adjusted odds ratios were calculated in order to analyse the six months compliance after the implementation. Multiple regression analyses was performed to control for age and gender in change of total scores in intervention and control groups.

## Results

### Compliance to the guidelines for depressive disorders

At baseline 122 patient records were included at the implementation clinics and 61 at the control clinics. There were no age or gender differences between patients from the intervention and control clinics. The percentages of patients being treated in accordance with each indicator at the implementation and control clinics are presented in Table 1, column 1 and 3. Some of the indicators were more often recorded in the implementation clinics; accessibility, diagnostic instrument, standardized rating scale initially and during treatment, substance/drug abuse and treatment plan. The total score differed significantly between implementation clinics and control clinics. Multiple linear regression analysis was used to compare change in total depression scores in intervention and control groups controlling for age and gender. The Beta value was significant for group (Beta = 0.54, p < 0.001), but not for age (Beta = -0.01, p < 0.9, or gender Beta = 0.08, p < 0.2).

**Table 1:** Percentages of patients being treated in accordance with each indicator at baseline before the implementation of clinical guidelines.

	Guidelines for depressive disorders			Guidelines for suicide attempters		
		
	Before Percentage, (n) implementation clinic/control clinic n = 122/61	Chisq, p-value	After Percentage, (n) implementation clinic/control clinic n = 121/60	Before Percentage, (n) implementation clinic/control clinic n = 121/61	Chisq, p-value	After Percentage, (n) implementation clinic/control clinic n = 120/60
Accessibility/wait time	77.9(95)/59.0(36)	7.1, 0.01	89.1(106)/53.3(32)	15.7(19)/29.5(18)	4.8, 0.1	14.2(17)/31.7(19)
Diagnostic assessment	83.6(102)/88.5(54)	ns	97.5(116)/90.0(54)	49.6(60)/26.2(16)	9.1, 0.01	73.3(88)/16.7(10)
Diagnostic instrument	12.3(15)/1.6(1)	5.7, 0.02	27.7(33)/0(0)	0(0)/0(0)	ns	7.5(9)/0(0)
Standardized rating scale	64.8(79)/44.3(27)	7.0, 0.01	91.6(109)/33.3(20)	41.3(50)/27.9(17)	3.2, 0.1	67.5(81)/16.7(10)
Standardized rating scale during treatment	50.0(61)/24.6(15)	10.8, 0.001	87.4(104)/38.3(23)	16.5(20)/16.4(10)	ns	52.5(63)/10.0(6)
Substance/drug abuse	46.7(57)/32.8(20)	3.2, 0.1	87.4(105)/53.3(32)	52.1(63)/55.7(34)	ns	64.2(77)/56.7(34)
Treatment (care) plan	59.8(73)/42.6(26)	4.8, 0.1	87.4(104)/38.3(23)	33.9(41)/44.3(27)	ns	67.5(81)/41.7(25)
Evaluation/outcome	66.4(81)/59.0(36)	ns	95.8(114)/55.0(33)	20.7(25)/19.7(12)	ns	47.5(57)/8.3(5)
Continuity	77.0(94)/78.7(48)	ns	95.0(113)/61.7(37)	86.0(104)/49.2(30)	28.2, 0.001	81.7(98)/31.7(19)
Suicide assessment	40.2(49)/45.9(28)	ns	95.8(115)/35.0(21)	55.4(67)/82.0(50)	12.5, 0.001	93.3(112)/73.3(44)
Antidepressant medication	54.1(66)/45.9(28)	ns	90.8(109)/36.7(22)			
Specialist assessment				50.4(61)/83.6(51)	18.9, 0.001	85.4(103)/83.3(50)
Follow-up				72.7(88)/75.4(46)	ns	88.3(106)/65.0(39)
Evaluation assessment				32.2(39)/18.0(11)	4.1, 0.1	64.2(77)/13.3(8)
	mean value (SD)	t-value, p-value		mean value (SD)	t-value, p-value	

Total score	6.3(1.7)/5.2(1.9)	3.9, 0.001	9.5(1.3)/5.0(1.5)	5.3(2.5)/5.3(2.2)	ns	8.1(2.3)/4.5(1.9)

At the six months follow-up 120 new patient records were included at the implementation clinics and 60 at the control clinics. There were no gender differences, but the mean age of the patients was slightly lower at the implementation clinics (35.4 years (SD 11.4) versus 38.6 years (SD 9.6), t = 1.9, df 178, p < 0.1). The only quality indicator that had an association with age was evaluation of outcome, which was less often registered in patients with higher age. The percentages and numbers of patients being treated in accordance with each indicator at follow-up are presented in Table 1, column 2 and 6. Age and gender adjusted odds ratios for the compliance for each depression indicator at the implementations clinics at the six months follow-up is presented in Table 2. The compliance was better in 8 of 11 depression indicators in the implementation group than the control group. For the indicator evaluation of outcome analyses were divided in different age groups (18–34 years (n = 125), 35–49 years (n = 80) and ≥50 years (n = 37)). In none of the three age groups the OR was significant. The results from the control clinics are presented as a comparison and only substance/drug abuse was significant. The mean score increased 3.4 in the implementation clinics and decreased 0.2 in the control clinics (t = 19.7, p < 0.001).

**Table 2:** The odds ratio of compliance six months after the implementation of clinical guidelines for the management of depression.

	Implementation clinics	Control clinics
Indicator	OR(CI_95%_)	OR(CI_95%_)

Accessibility/wait time	2.2(1.0–4.7)	0.6(0.3–1.4)
Diagnostic assessment	7.9(2.3–27.8)	1.2(0.4–3.6)
Diagnostic instrument	2.7(1.4–5.4)	*
Standardized rating scale	6.2(2.9–13.2)	0.7(0.3–1.4)
Standardized rating scale during treatment	7.1(3.7–13.7)	1.9(0.9–4.1)
Substance/drug abuse	8.1(4.2–15.7)	2.9(1.3–6.6)
Treatment (care) plan	4.9(2.5–9.5)	0.9(0.4–1.9)
Evaluation/outcome	12.4(4.7–32.9)	0.8(0.4–1.7)
Continuity	5.6(2.2–14.2)	0.4(0.2–1.0)
Suicide assessment	34.7(13.2–91.4)	0.6(0.3–1.3)
Antidepressant medication	8.5(4.2–17.5)	0.7 (0.3–1.4)
	t-value, p-value	t-value, p-value

Total score	16.2, 0.001	ns

*The numbers did not allow calculations.

### Compliance to the guidelines for the management of suicide attempters

At baseline 121 patient records were included at the implementation clinics and at the control clinics 61 records. There were no gender differences but the patients at the implementation clinics were slightly younger (32.5 years (8 SD 12.2) versus 38.3 years (SD 15.1), t = 2.8, df 180, p < 0.01). The only indicator that differed in registration was continuity of care giver that was less often recorded in older patients. The differences between the implementation clinics and the control clinics in the documentation of the quality indicators are presented in Table 1, column 1 and 3. Some of the indicators were more often recorded in the implementation clinics, diagnostic assessment, standardized rating scale initially, evaluation and evaluation assessment. Others were more frequently recorded at the control clinics, accessibility, substance/drug abuse, suicide and specialist assessment. The mean score did not differ between implementation clinics and control clinics. For continuity of care giver separate analyses were made in three age-groups (18–34, 35–49 and ≥50 years). There were differences between the implementation and control clinic in the in the two younger age-groups but not in the oldest (Chisq 12.4, df 1, p < 0.001, Chisq 19.0, df 1, p < 0.01, ns)

At the six months follow-up 120 patient records were included at the implementation clinics and at the control clinics 60 records. There were no age differences, but the patients at the implementation clinics were more often females (70.8% versus 58.3%, chisq = 2.8, df 1, p < 0.1)). The only quality indicator that had an association with gender was specialist assessment, which was less often registered in females. The percentages and numbers of patients being treated in accordance with each indicator at follow-up is presented in Table 1, column 2 and 6. Age and gender adjusted odds ratios for the compliance for each indicator at the implementations clinics at the six months follow-up are presented in Table 3. For the indicator specialist assessment analyses were divided according to gender. In both gender the OR was significant; females: 3.4(1.7–6.9), males 33.0(6.8–161.3). The results from the control clinics are presented as a comparison and none of the OR was significant. The mean score increased 2.8 in the implementation clinics and decreased 0.8 in the control clinics (t = 9.5, p < 0.001). A similar procedure as for comparing total depression score was performed for total suicide scores and the Beta value was significant for group (Beta = 0.3, p < 0.001, but not for age (Beta = -0.04, p < 0.4, or gender Beta = 0.03, p < 0.5).

**Table 3:** The odds ratio of compliance six months after the implementation of clinical guidelines for the management of suicidal patients.

	Implementation clinics	Control clinics
Indicator	OR(CI_95%_)	OR(CI_95%_)

Accessibility/wait time	0.9(0.4–1.8)	1.1(0.5–2.4)
Diagnostic assessment	2.8(1.6–4.8)	0.6(0.2–1.5)
Diagnostic instrument	*	*
Standardized rating scale	2.9(1.7–4.9)	0.5(0.2–1.4)
Standardized rating scale during treatment	6.0(3.3–11.2)	0.6(0.2–1.9)
Substance/drug abuse	1.7(1.0–2.8)	1.0(0.5–2.2)
Treatment (care) plan	4.6(2.6–8.1)	0.9(0.4–2.0)
Evaluation/outcome	3.5(2.0–6.2)	0.4(0.1–1.2)
Continuity	0.7(0.4–1.5)	0.5(0.2–1.0)
Suicide assessment	11.5(5.1–25.8)	0.6(0.3–1.5)
Specialist assessment	6.6(3.5–12.6)	0.8(0.3–2.3)
Follow-up	2.9(1.4–5.7)	0.6(0.3–1.4)
Evaluation assessment	3.9(2.2–6.4)	0.7(0.3–1.8)
	t-value, p-value	t-value, p-value

Total score	9.0, 0.001	-2.1, 0.1

*The numbers did not allow calculations.

## Discussion

This study showed that six months compliance to clinical guidelines for the treatment of depression and the management of suicide attempters measured by indicators of required clinical practice was enhanced by an active implementation. The clinics, to which the guidelines were only disseminated, showed no improvement.

A multifaceted intervention including several active strategies is more likely to be effective than a single active strategy [21]. Interactive approaches, such as audit, feedback, academic detailing seems most effective at changing physician care and patient outcome, but are insufficient by them self [22]. Shortell et al. [23] suggested that four dimensions are needed for a successful implementation, i.e. process, strategic, cultural, technical and structural. Our implementation programme included all of those. The programme consisted of the introduction of regional mandatory evidence-based clinical guidelines for psychiatric disorders in Stockholm County, a top-down strategic initiative. The programme encouraged the formation of local teams at the clinics which created interest, engagement and motivation, giving rise to a culture positive to guidelines implementation. We acted on the knowledge that without an appropriate organizational culture, only small, temporary improvements will be possible [23]. Measurements were based on indicators derived from the guidelines' evidence based requirements of preferred practice, giving the implementation a clear structure. The use of the indicators enabled regular feedback on gaps in performance, compared to guidelines, which was strengthened by outreach visits by the researcher, an expert on the guidelines. Conditions for a learning process were created. These elements of the programme are strategies previously reported to be useful in implementation [10]. A summary of the implementation programme is provided in Table 4.

**Table 4:** The four dimensions of the implementation process at intervention and control clinics.

	Strategic	Cultural	Technical	Structural
Intervention clinics	Mandatory clinical guidelines, local implementation teams with expert feed-back and support	Local groups formed a developmental culture by following-up their process. Engagement, teamwork and participation.	Indicators used as identifiers of need for change, measures of change, audits	Facilitated learning. Seminars, regular academic detailing
Control clinics	Mandatory clinical guidelines	No groups	Indicators only mentioned in the guidelines	None of the above

Strategic × cultural × technical × structural = Result

The process indicators used in this study could also be used to assess the quality of the care documented in patient records. Analyses of the documentation in this study showed that the indicators had a high inter-rater reliability and were easy to use. The study also showed that the indicators were feasible for audit and feedback as part of the implementation strategy. The used indicators are objective measures that can be used by clinicians, managers and the public when assessing the quality of the process and the outcome of patient care [24,25]. Hermann and Palmer [26] have argued that process measures may well be used more effectively in metal health care.

In a previous study antidepressant dosage and duration adequacy have been used as guideline based process indicators to identify if depression care was based on clinical guidelines [27]. Indicators can be useful in determine the maintenance of a good standard of psychiatric service, and in detecting and stimulate solutions to problems found in service delivery.

This is a small study and the results are based on patient records and process quality scores only. Therefore, the study needs to be replicated to confirm that random change and selection bias have not combined to produce a spurious result. We plan to do this using other clinics in a near future. The intervention clinics had all volunteered to participate and, thus, they probably consisted of the most motivated and quality-focused in the region, potentially introducing a bias. The fact that indicator documentation for depression patients at base-line was more frequent among intervention clinics supports that assumption. When managing suicide attempters the picture at baseline was mixed, with some indicators more frequently documented in control clinics.

Another limitation was that the study relied on self-reporting. Local teams at the clinics assessed the patient records for indicators. However, a supervisor (the first author) paid regular visits to the clinic, discussing the registration. A random sample of records was reassessed by the first author revealing an acceptable inter-rater reliability (Kappa 0.92–1.0).

The strengths of the study are its longitudinal nature and the fact that it was a quasi-experimental involving measures before-and-after the intervention with a comparison group. However, the follow-up was only six months and the long-term impact is unknown.

This implementation model, although promising in the light of our study, needs to be further tested. In this study we show that these indicators were easy to use and had high inter-rater reliability. As indicated in the literature, they can also be used for quality assessment. The challenge in all implementation work is to achieve improvement, sustainable over time. We plan to follow-up the studied clinics to learn more about their guidelines compliance and the effects of using the indicators systematically. The experiences gained from the local implementation will contribute to the modification, revision and adaptation of the guidelines, further enhancing their perceived usefulness and local application.

## Conclusion

The findings in this study suggest that compliance to clinical guidelines measured by indicators of required clinical practice was enhanced by an active implementation. The active implementation included four dimensions: strategic, cultural, technical and structural.

## Competing interests

The authors declare that they have no competing interests.

## Authors' contributions

TF, AÅW, MB and YF have all participated to the design of the study. TF and YF have analyzed the data. All authors read and approved the final manuscript.

## Pre-publication history

The pre-publication history for this paper can be accessed here:


